# Network-Pharmacology-Based Study on Active Phytochemicals and Molecular Mechanism of *Cnidium monnieri* in Treating Hepatocellular Carcinoma

**DOI:** 10.3390/ijms23105400

**Published:** 2022-05-12

**Authors:** Shakeel Ahmad Khan, Terence Kin Wah Lee

**Affiliations:** Department of Applied Biology and Chemical Technology, The Hong Kong Polytechnic University, 11 Yuk Choi Rd., Hung Hom, Kowloon 999077, Hong Kong

**Keywords:** *Cnidium monnieri*, network pharmacology, active phytochemicals, molecular mechanism, hepatocellular carcinoma

## Abstract

Hepatocellular carcinoma (HCC) is a malignancy with a high mortality rate globally. For thousands of years, *Cnidium monnieri* has been used to treat human ailments and is regarded as a veritable treasure trove for drug discovery. This study has investigated the key active phytochemicals and molecular mechanisms of *Cnidium monnieri* implicated in curing HCC. We utilized the TCMSP database to collect data on the phytochemicals of *Cnidium monnieri*. The SwissTargetPrediction website tool was used to predict the targets of phytochemicals of *Cnidium monnieri*. HCC-related genes were retrieved from OncoDB.HCC and Liverome, two liver-cancer-related databases. Using the DAVID bioinformatic website tool, Gene Ontology (GO) and KEGG enrichment analysis were performed on the intersecting targets of HCC-related genes and active phytochemicals in *Cnidium monnieri*. A network of active phytochemicals and anti-HCC targets was constructed and analyzed using Cytoscape software. Molecular docking of key active phytochemicals was performed with anti-HCC targets using AutoDock Vina (version 1.2.0.). We identified 19 active phytochemicals in *Cnidium monnieri*, 532 potential targets of these phytochemicals, and 566 HCC-related genes. Results of GO enrichment indicated that *Cnidium monnieri* might be implicated in affecting gene targets involved in multiple biological processes, such as protein phosphorylation, negative regulation of the apoptotic process, which could be attributed to its anti-HCC effects. KEGG pathway analyses indicated that the PI3K–AKT signaling pathway, pathways in cancer, proteoglycans in cancer, the TNF signaling pathway, VEGF signaling pathway, ErbB signaling pathway, and EGFR tyrosine kinase inhibitor resistance are the main pathways implicated in the anti-HCC effects of *Cnidium monnieri*. Molecular docking analyses showed that key active phytochemicals of *Cnidium monnieri*, such as ar-curcumene, diosmetin, and (E)-2,3-bis(2-keto-7-methoxy-chromen-8-yl)acrolein, can bind to core therapeutic targets EGFR, CASP3, ESR1, MAPK3, CCND1, and ERBB2. The results of the present study offer clues for further investigation of the anti-HCC phytochemicals and mechanisms of *Cnidium monnieri* and provide a basis for developing modern anti-HCC drugs based on phytochemicals in *Cnidium monnieri*.

## 1. Introduction

Hepatocellular carcinoma (HCC) is the fifth most prevalent and high-mortality-rate malignancy globally [1,2]. HCC is a multifactorial ailment intimately associated with persistent viral infection, toxin neoplasia, cirrhosis caused by fatty liver disease or alcoholism, and hereditary factors [1]. Numerous therapeutic approaches are being explored for treating HCC, including systemic sorafenib treatment, liver transplantation, transarterial chemoembolization, local ablation, and surgical resection [3]. Sorafenib is the most commonly used medicine to treat HCC. It works by inhibiting protein kinases such as RAF, VEGFR, and PDGFR. However, because most people with HCC are diagnosed in an advanced phase, sorafenib treatment remains the only effective contemporary choice [4]. Additionally, fewer than 20% of individuals tolerate sorafenib adequately, resulting in frequent and severe adverse effects [5]. Consequently, it has become obligatory to develop more efficacious and less toxic alternative medicines to enhance the survival rates of HCC patients. 

One promising alternative is Chinese herbal medicine (CHM) for treating HCC. Thousands of years have passed since CHM was first employed therapeutically. Due to its comprehensive scientific assessment in basic research and clinical trials, CHM is considered a treasure trove for alternate antineoplastic medicine development [6,7]. Reports demonstrate that phytochemicals of some herbaceous plants have promising capabilities to impede cell proliferation, inhibit carcinogenesis, and prevent tumor metastasis very efficiently [8,9]. Compared with conventional chemotherapeutic drugs, their selectivity in killing melanoma cells and their low toxicity to healthy cells make them more attractive as cancer treatment alternatives [10]. 

*Cnidium monnieri* (L.) Cuss., (She Chuang Zi in Chinese) is a Chinese herbal remedy that has been used for over 2000 years. It originated with Shennong’s *Classic of Materia Medica* (Shennong Bencao Jing), which was composed during the Eastern Han Dynasty (25–220 AD). *Cnidium monnieri* has historically been used to treat female vaginal problems, male impotence, and skin ailments [11]. In contemporary TCM therapeutic practice, water decoctions and tinctures of *Cnidium monnieri* are often used alone or in conjunction with other Chinese medicinal herbs (Sophora flavescens, Phellodendron amurense, etc.) to treat persistent skin itch, superficial fungal infections, and atopic dermatitis [12,13]. Over 400 phytochemicals, including glucides, glycosides, terpenoids, monoterpenoid glucosides, chromones, liposoluble compounds, volatile oils, and coumarins, have been isolated and identified in *Cnidium monnieri* [14,15]. *Cnidium monnieri* extracts and components have been found in pharmacological investigations to possess antibacterial, anticancer, antitumor, and anti-inflammatory properties [12,15], which may be used to prevent and cure liver infections caused by hepatitis and HCC. Osthole, 7-methoxy-8-(3-methyl-2-butenyl) coumarin, is a simple bioactive coumarin derivative isolated from *Cnidium monnieri* that has been shown to suppress HCC growth and induce apoptosis [10]. However, the anti-HCC compounds and pathways by which *Cnidium monnieri* treats HCC remain unknown. 

**Figure 1:**
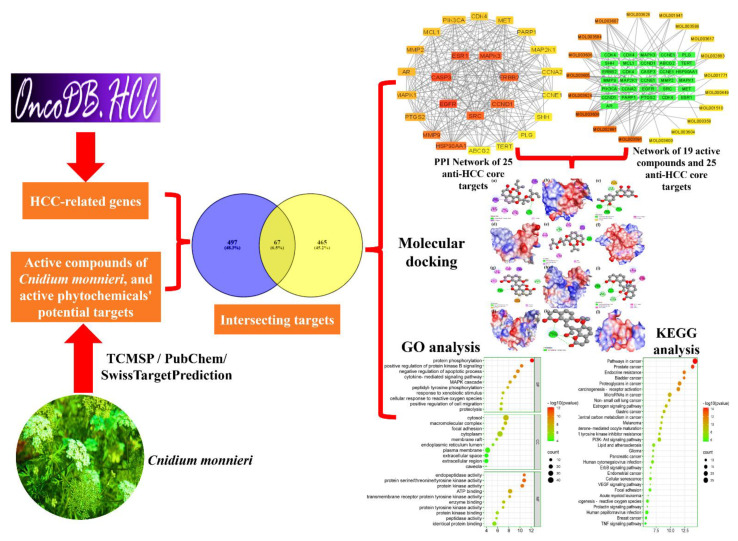
Flowchart of the current research study.

Thus, the scientists intend to offer a theoretical foundation for discovering and developing novel drugs by examining the anti-HCC compounds and associated molecular mechanisms of dried *Cnidium monnieri* fruits in treating HCC. Network pharmacology is a relatively emerging multidisciplinary field of drug research that utilizes Big Data and artificial intelligence [16]. It is extensively employed to identify active pharmaceutical ingredients and understand drugs’ overall mechanism of action, therefore offering innovative technical and scientific support for novel drug research and development and clinical medication usage [17]. Compared with Western medicine, which is based on a single drug for a single target, CHM delivers its therapeutic and pharmacological effects as a whole via multiple targets and multiple components. Based on CHM’s holistic approach, network pharmacology aims to explore drugs’ efficacy on a holistic level, thus transitioning the research approaches away from the established one drug, one target model, and toward a developing one drug, network targets mode [18,19,20]. This technique is promising for elucidating the pathways by which CHMs have synergistic effects in cancer therapy [21]. Currently, a number of researchers are utilizing network pharmacology to decipher the molecular mechanisms of phytomolecules implicating the cure of various diseases [22,23]. Therefore, in this research, we employed network pharmacology and bioinformatics tools to predict biologically active phytomolecules, target proteins, and molecular pathways in *Cnidium monnieri* to treat HCC. The findings were further validated using molecular docking and text mining. To the best of the author’s information, this is the first report to use network pharmacology to predict the active phytochemicals and molecular mechanisms of *Cnidium monnieri* in treating HCC; Figure 1 depicts the flowchart of the current research study.

## 2. Results

### 2.1. Screening of Active Phytochemicals in Cnidium monnieri

A total of 114 phytochemicals of *Cnidium monnieri* were retrieved from the TCMSP [24]. Nineteen active phytochemicals in *Cnidium monnieri* were obtained based on oral bioavailability (OB) ≥ 30% and drug-likeness (DL) ≥ 0.18. These active phytochemicals are given in Table 1 with their structures, OB, and DL.

### 2.2. Active Phytochemicals Targets 

**Figure 2:**
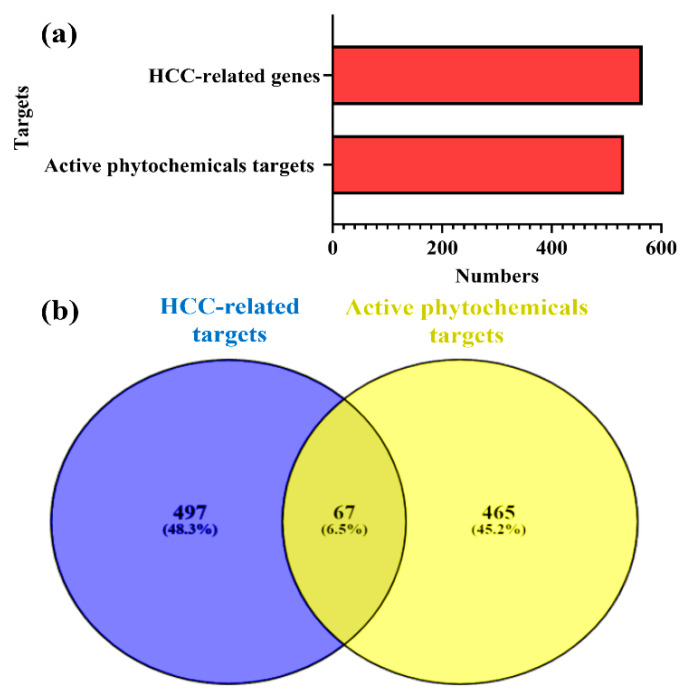
(**a**) Potential protein targets of active phytochemicals in *Cnidium monnieri* and HCC-related genes. (**b**) Intersecting targets between the active phytochemical potential protein targets in *Cnidium monnieri* and HCC-related genes determined by VENNY 2.1.

The SwissTargetPrediction database was employed for determining potential protein targets for the active phytochemicals in *Cnidium monnieri* [25]. A total of 1387 potential protein targets were obtained with a probability score > 0. After the removal of redundancies, 532 potential protein targets of active phytochemicals in *Cnidium monnieri* were investigated further (Figure 2a).

### 2.3. HCC-Related Genes

A total of 564 HCC-related genes were recovered from OncoDB.HCC (http://oncodb.hcc.ibms.sinica.edu.tw, accessed on 10 March 2022) and Liverome (http://liverome.kobic.re.kr/index.php, accessed on 10 March 2022) (Figure 2a) [1,26,27].

### 2.4. Intersecting Targets between HCC-Related Genes and Potential Protein Targets of Active Phytochemicals

Intersecting targets were recognized between the HCC-related genes and potential protein targets of active phytochemicals using the VENNY 2.1.0. online system [28]. A total of 67 intersecting targets were identified between them, as presented in Figure 2b.

### 2.5. Network Construction and PPI Analysis

**Figure 3:**
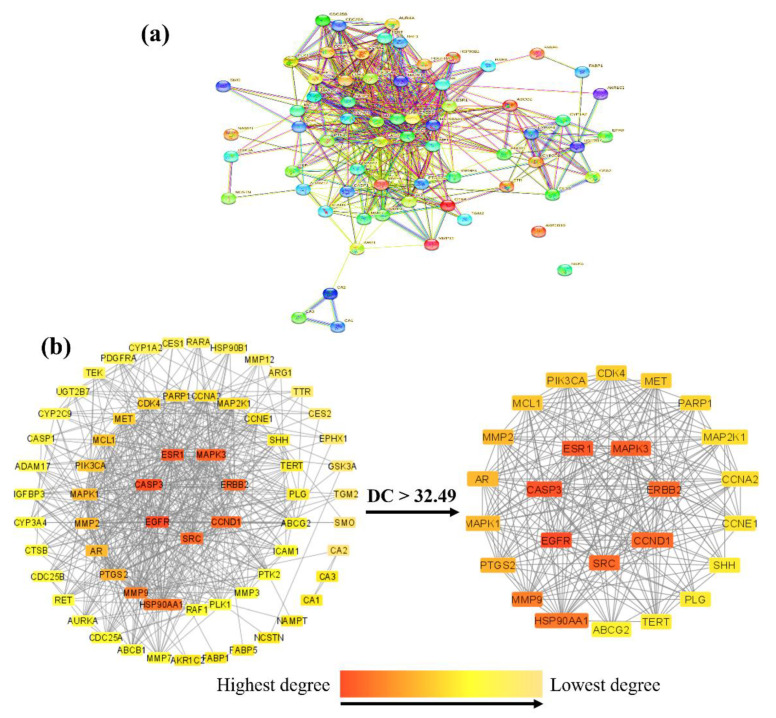
(**a**) PPI network of 67 intersecting targets constructed with STRING. (**b**) The PPI network of the 67 intersecting targets and the 25 potential anti-HCC core targets was constructed using Cytoscape software. Each node’s color denotes the degree, from red (highest) to yellow (lowest), as the node degree decreases.

The PPI network was constructed by importing the 67 intersecting targets to the STRING database [29]. The PPI network contained 67 nodes and 528 edges (Figure 3a). The average PPI enrichment *p*-value, average local clustering coefficient, and average node degree were *p* < 0.00001, 0.609, and 15.8, respectively. The STRING PPI results were further analyzed by exporting them in a simple textual data format (.tsv) file to Cytoscape software (version 3.9.0) [30]. The results showed that the PPI network involved 65 nodes and 1056 edges. The characteristic path length between all node pairs was 2.045. The PPI network radius, diameter, heterogeneity, and density were 3, 5, 0.682, and 0.254, respectively. Figure 3b shows the PPI network with colored nodes; the color of each node denotes the degree, from red (highest) to yellow (lowest), as the node degree decreases.

**Figure 4:**
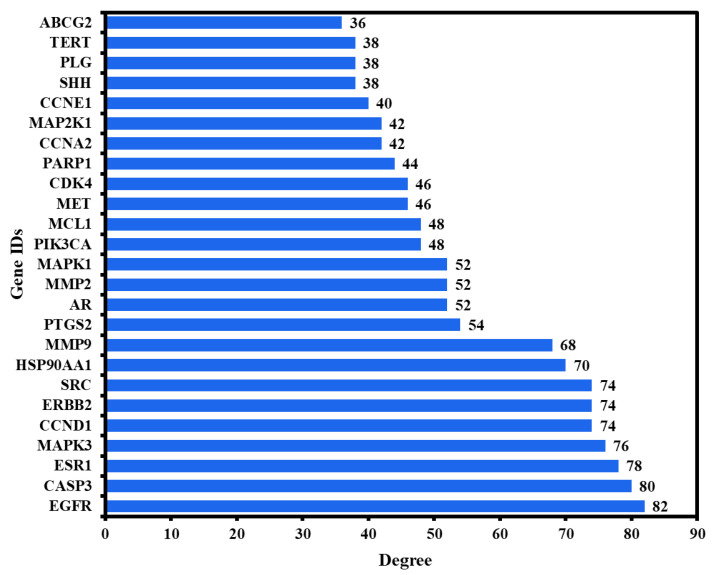
The PPI network’s 25 potential anti-HCC core targets ranked by DC ≥ average value of 32.49.

The 25 nodes that achieved the degree centrality (DC) criterion with an average value greater than 32.49 were further extracted and classified as potential anti-HCC core targets (Figure 3b). The 25 potential anti-HCC core targets, ranked according to DC, are depicted in Figure 4 as a bar graph. The top 6 potential anti-HCC core targets, i.e., EGFR, CASP3, ESR1, MAPK3, ERBB2, and CCND1, were chosen for molecular docking studies, with key active phytochemicals described in Section 2.6.

### 2.6. Network of Active Phytochemicals and Anti-HCC Targets

**Figure 5:**
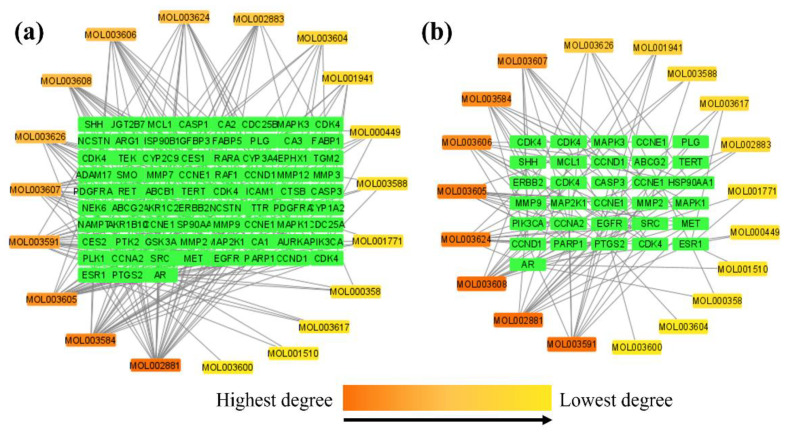
(**a**) Network between the 19 active phytochemicals and 67 intersecting targets. (**b**) Hub network between the 19 active phytochemicals and 25 potential anti-HCC core targets. Each node’s color denotes the degree, from red (highest) to yellow (lowest), as the node degree decreases.

A network depicting the 19 active phytochemicals (as described in Section 2.1) interacting on the 67 intersecting targets (as described in Section 2.4) was developed using Cytoscape 3.9.0 software (Figure 5a). The network consisted of 94 nodes and 203 edges, as shown by the results. Each edge represents the interaction of active phytochemicals with intersecting targets. Each node of phytochemicals is colored according to their degree, ranging from red (highest) to yellow (lowest) as the degree of the node decreases. The node degree in a network denotes the number of edges that link it to other nodes [31].

**Figure 6:**
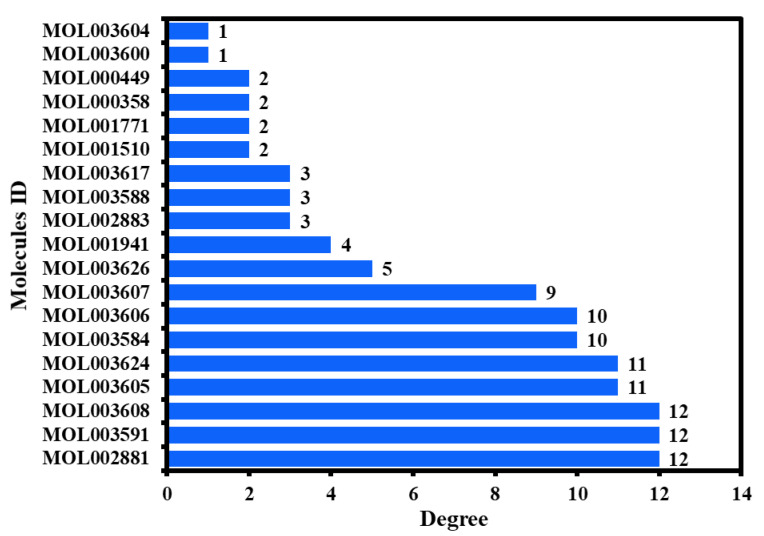
The 19 active phytochemicals based on their degrees in the hub network.

Furthermore, as seen in Figure 5b, a hub network was constructed between the 19 active phytochemicals and 25 potential anti-HCC core targets (Section 2.5). Each of the 19 active phytochemicals acted on the 25 potential anti-HCC core targets. The 19 active phytochemicals are shown in Figure 6 as a bar graph based on their degrees in the hub network.

Ranked by DC ≥ 6.025 (average value) in the hub network, the eight active phytochemicals acting on more than eight targets were MOL002881 (diosmetin), MOL003591 (ar-curcumene), MOL003608 (O-acetylcolumbianetin), MOL003605 [(E)-2,3-bis(2-keto-7-methoxy-chromen-8-yl)acrolein], MOL003624 (O-isovalerylcolum bianetin), MOL003584 (xanthoxylin N), MOL003606 (cniforin A), and MOL003607 (cniforin B) (Figure 6). These eight active phytochemicals are considered the most important and key active phytochemicals found in *Cnidium monnieri* for HCC treatment. The network findings reveal that a single phytochemical may interact with multiple HCC targets, and multiple phytochemicals can interact with a single HCC target. These results emphasize the intricacy of the interactions between multiple targets and active phytochemicals in *Cnidium monnieri*.

### 2.7. GO Enrichment Analysis

**Figure 7:**
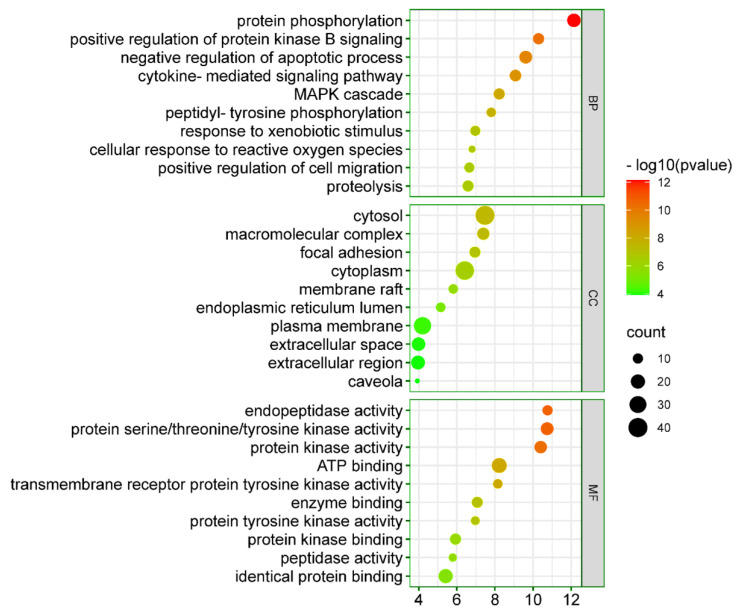
GO enrichment analysis of the 67 intersecting targets for the treatment of HCC with *Cnidium monnieri*.

GO enrichment analysis was performed on 67 intersecting targets employing DAVID web-based tool (Version 6.8) [32]. The 239 BP, 42 CC, and 59 MF terms were found to meet the screening threshold of *p* ≤ 0.05. The top 10 enrichment terms of BP, CC, and MF are presented in a bubble diagram (Figure 7). The results of GO enrichment analysis indicated that the gene targets are implicated in multiple BPs, such as protein phosphorylation, positive regulation of protein kinase B signaling, negative regulation of the apoptotic process, cytokine-mediated signaling pathway, MAPK cascade, proteolysis, positive regulation of cell migration, etc. In the enriched CC category, gene targets are implicated in the cytosol, cytoplasm, plasma membrane, extracellular region, extracellular space, macromolecular complex, etc. GO enrichment analysis results showed that the enriched MF ontologies are dominated by ATP binding, identical protein binding, proteins kinase activity, protein serine/threonine kinase activity, enzyme binding, proteins kinase binding, etc.

### 2.8. KEGG Pathways Analysis

**Figure 8:**
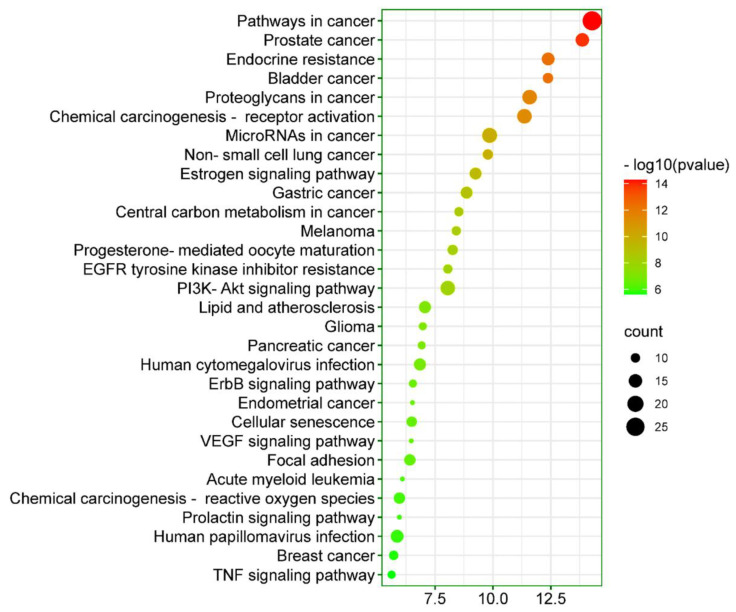
The top 30 enriched KEGG pathways of 67 intersecting targets involved in *Cnidium monnieri*’s anti-HCC therapeutic effects.

KEGG pathway analysis was performed on 67 intersecting targets to elucidate the molecular mechanism by which *Cnidium monnieri* treats HCC using the DAVID web-based tool (Version 6.8) [32]. A total of 117 enriched KEGG pathways were found to meet the screening threshold of *p* ≤ 0.05. The top 30 enriched KEGG pathways were further presented in bubble plot form, as displayed in Figure 8. The results of the KEGG pathway enrichment analysis showed that the molecular mechanisms by which *Cnidium monnieri* treats HCC may be implicated in pathways in cancer, proteoglycans in cancer, chemical carcinogenesis receptor activation, microRNAs in cancer, PI3K-AKT signaling pathway, estrogen signaling pathway, TNF signaling pathway, VEGF signaling pathway, ErbB signaling pathway, EGFR tyrosine kinase inhibitor resistance, etc. These signaling pathways could all function together in *Cnidium monnieri*’s anti-HCC therapeutic effects.

### 2.9. Molecular Docking

**Figure 9:**
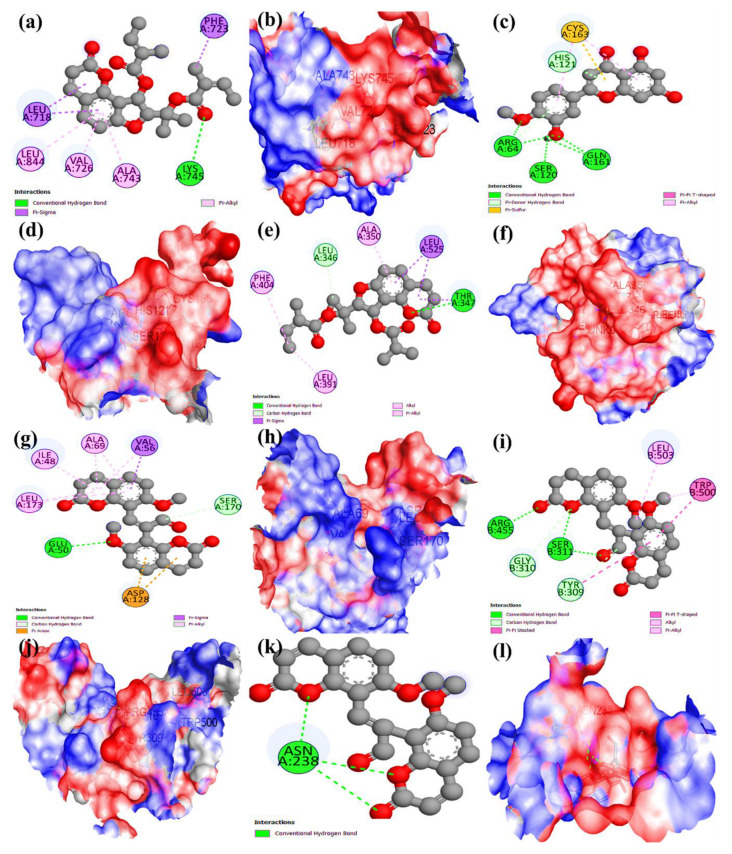
Molecular docking results of key active phytochemicals with anti-HCC core targets. (**a**,**b**) (**2D and 3D**) MOL003591 binds with EGFR. (**c**,**d**) (**2D and 3D**) MOL002881 binds with CASP3. (**e**,**f**) (**2D and 3D**) MOL003607 binds with ESR1. MOL003605 binds with (**g**,**h**) (**2D and 3D**) MAPK3, (**i**,**j**) (**2D and 3D**) CCND1, and (**k**,**l**) (**2D and 3D**) ERBB2.

The molecular docking of eight key active phytochemicals of *Cnidium monnieri* (as determined in Section 2.6) with six anti-HCC core targets (as determined in Section 2.5) was performed. The findings are summarized in Table 2. The docked complexes that showed the best binding affinities are shown in Figure 9a–l. The lower the binding energy of the phytochemicals, the greater their affinity for the targets (proteins). The molecular docking results demonstrated that all key active phytochemicals of *Cnidium monnieri* could bind with EGFR, CASP3, ESR1, MAPK3, and CCND1 with strong binding affinity. However, they had a moderate binding affinity with ERBB2. Among eight active phytochemicals, Ar-curcumene, and (E)-2,3-bis(2-keto-7-methoxy-chromen-8-yl)acrolein presented excellent binding affinity with EGFR with the least energy score < −8.0. The phytochemicals Diosmetin, (E)-2,3-bis(2-keto-7-methoxy-chromen-8-yl)acrolein, and Ar-curcumene showed a greater affinity for CASP3 and exhibited the least energy score < −6.0 compared with others. Cniforin B and O-isovalerylcolum Bianetin demonstrated excellent binding affinity to ESR1 and had the lowest energy score, < −8.5, as compared with other phytochemicals. The phytochemical (E)-2,3-bis(2-keto-7-methoxy-chromen-8-yl)acrolein binds to MAPK3 and CCDN1 with higher affinity, with −8.1 and −8.2 energy binding scores, respectively. (E)-2,3-bis(2-keto-7-methoxy-chromen-8-yl)acrolein has a modest binding affinity for ERBB2 with an energy score of −4.3.

The molecular docking findings reveal that eight of *Cnidium monnieri*’s key active phytochemicals have protective effects on the anti-HCC core targets (EGFR, CASP3, ESR1, MAPK3, CCND1, and ERBB2). The docking results corroborated the network pharmacology screenings, thus validating the reliability of network pharmacology in this investigation.

## 3. Discussion

Hepatocellular carcinoma is a malignancy with a high mortality rate. For thousands of years, CHMs have been used to treat human ailments and are regarded as a veritable treasure trove for drug discovery [1,33]. This study investigated the key active phytochemicals and molecular mechanisms of *Cnidium monnieri* implicated in HCC treatment. *Cnidium monnieri* is composed of complex systems of multiple phytochemicals. The phytochemicals that satisfied the criteria of OB ≥ 30% and DL ≥ 0.18 were regarded as biologically active in *Cnidium monnieri*. A total of 19 phytochemicals in *Cnidium monnieri* were found to be active in treating HCC. Most of the active phytochemicals in *Cnidium monnieri* were found to be multitargeting. Network results of active phytochemicals and anti-HCC targets demonstrated the key active phytochemicals which interact with more than eight anti-HCC core targets are MOL002881 (diosmetin), MOL003591 (ar-curcumene), MOL003608 (O-acetylcolumbianetin), MOL003605 [(E)-2,3-bis(2-keto-7-methoxy-chromen-8-yl)acrolein], MOL003624 (O-isovalerylcolum bianetin), MOL003584 (xanthoxylin N), MOL003606 (cniforin A), and MOL003607 (cniforin B). The results demonstrate that eight key active phytochemicals of *Cnidium monnieri* modulated most of the HCC targets and exhibited immunosuppressive activity. Hence, we can deduce that key active phytochemicals might have a synergistic effect in treating HCC.

The PPI network analysis indicated that multiple genes (EGFR, CASP3, ESR1, MAPK3, CCND1, and ERBB2) are associated with the effects of *Cnidium monnieri* on HCC treatment. Reports demonstrate that the aberrant expression of EGFR has the leading function in causing HCC etiology. The upregulation of EGFR has been reported in liver macrophages in both human and animal HCC models, where it functions as a tumor promoter [34,35]. HCC is often accompanied by hepatocyte apoptosis and adaptive proliferation. However, the involvement of CASP3, an apoptotic pathway effector cysteine protease, in hepatocarcinogenesis is not well understood, with conflicting evidence of its expression [36]. Persad et al. reported that frequent overexpression of CASP3 contributes to the development of HCC [37]. Therefore, regulating EGFR and CASP3 expression can be a viable therapeutic approach. ESR1 has been identified as a tumor suppressor gene, with hypermethylation of the promoter correlated with tumor growth. Its expression was found to negatively interact with the size and stage of HCC tumors in a genome-wide expression study, commensurate with preclinical findings revealing that ESR1 deficiency promotes tumorigenesis and the progression of HCC [38,39,40,41]. MAPK3 is a key component of the MAPK pathway and has a high prognostic effect on individuals with HCC. MAPK3 is required for the ERK signaling pathway to function correctly. It governs cell proliferation, cycling, and apoptosis, and its overexpression has been found in human HCC cells [42,43]. The CCND1 subunit of holoenzyme is implicated in the phosphorylation and inactivation of the retinoblastoma protein. Overexpression of CCND1, an oncogene, contributes to poor prognosis and tumor recurrence, leading to the etiology of HCC [44,45]. ERBB2 belongs to a family of epidermal growth factor receptors that contributes to the differentiation signals and transmission of proliferation. ERBB2 expression was shown to be high in 30% to 40% of HCCs in various investigations [46,47].

The GO enrichment analysis revealed that *Cnidium monnieri* might be implicated in affecting gene targets that are involved in multiple BPs (protein phosphorylation, negative regulation of the apoptotic process, positive regulation of protein kinase B signaling, cytokine-mediated signaling pathway, MAPK cascade, proteolysis, positive regulation of cell migration, etc.) which could be attributed to its anti-HCC effects (Figure 7). Protein phosphorylation is required for protein function, cellular localization, and biological processes. However, the aberrant phosphorylation of proteins results in a variety of medical conditions, including HCC [48]. Apoptosis is a physiological process of eliminating superfluous cells throughout liver growth and regeneration; however, insufficient apoptosis leads to the development and progression of tumors in the biliary tree and liver. Defective apoptosis might result due to the overactivation of anti-apoptotic pathways (negative regulation of the apoptotic process) [49,50]. Moreover, protein kinase B signaling stimulates a variety of biological activities, including apoptosis, glucose metabolism, cell proliferation, transcription, and migration; however, its overexpression has been linked to malignancies, including HCC [51,52]. The enriched CC further indicated that the gene targets are implicated in the cytosol, cytoplasm, plasma membrane, extracellular region, extracellular space, macromolecular complex, etc. GO enrichment analysis results showed that the enriched MF ontologies are dominated by gene targets implicated in ATP binding, identical protein binding, proteins kinase activity, protein serine/threonine kinase activity, enzyme binding, proteins kinase binding, etc.

The KEGG pathway enrichment analysis revealed that the molecular mechanisms by which *Cnidium monnieri* treats HCC might be implicated in pathways in cancer, proteoglycans in cancer, the PI3K–AKT signaling pathway, estrogen signaling pathway, TNF signaling pathway, VEGF signaling pathway, ErbB signaling pathway, EGFR tyrosine kinase inhibitor resistance, etc. (Figure 8). Twenty-seven targets are enriched in pathways in cancer. Reports demonstrate that the upregulation of proteoglycans such as glypican-3 significantly contributes to the pathogenesis of several melanoma types. However, their highest positive case rates have been observed in HCC patients among all cancer types [53]. Thus, suppressing the proteoglycans can be a viable therapeutic opportunity in treating HCC. Although the PI3K–AKT signaling pathway modulates several cellular activities, including differentiation, metabolism, survival, and apoptosis, its aberrant activation contributes to developing HCC malignancies [54]. Thus, regulating the activation of PI3K-AKT may be another therapeutic option for HCC treatment. TNF signaling promotes hepatocyte apoptosis in the liver, resulting in liver injury and indirectly contributing to carcinogenesis through a variety of inflammatory processes such as chronic viral hepatitis [55,56]. VEGF signaling is a potent HCC cell motility, infiltration, and angiogenesis activator. Therefore, VEGF inhibition may be a potential strategy in the therapy of HCC. [57]. Studies have reported that the overexpression of EGFR in the liver contributes to poor prognosis, rapid proliferation, and metastasis. Thus, suppressing EGFR signaling is a potential and feasible opportunistic strategy for treating HCC [58]. These signaling pathways could all function together in the molecular mechanisms of *Cnidium monnieri* in treating HCC. Molecular docking analysis further corroborated that *Cnidium monnieri*’s active phytochemicals had protective effects on HCC-related targets (EGFR, CASP3, ESR1, MAPK3, CCND1, and ERBB2).

## 4. Materials and Methods

### 4.1. Collection of Active Phytochemicals in Cnidium monnieri

We employed the TCMSP (Version 2.3, https://old.tcmsp-e.com/tcmsp.php, accessed on 10 December 2021) to collect all herbal medicinal phytochemicals of *Cnidium monnieri* [24]. TCMSP is a novel pharmacology analysis tool that helps find new drugs from herbal medicines. The criteria of drug-likeness (DL) and oral bioavailability (OB), such as ≥ 0.18 and ≥30%, respectively, were utilized to obtain potential active phytochemicals of *Cnidium monnieri*. These two critical elements significantly contribute to a substance’s pharmacological ability [1].

### 4.2. SwissTargetPrediction of Active Phytochemicals of Cnidium monnieri

The SwissTargetPrediction database (http://www.swisstargetprediction.ch/, accessed on 10 December 2021) was used to identify potential protein targets for active phytochemicals in *Cnidium monnieri* with limitations to “Homo sapiens” [25]. The SwissTargetPrediction database gives results of a maximum of 100 probable protein targets for each phytochemical. These 100 probable protein targets are ranked based on their probability score, ranging from 0 to 1. We have selected potential protein targets with a probability score > 0.

### 4.3. Screening of HCC-Related Genes

HCC-related genes were salvaged from the OncoDB.HCC (http://oncodb.hcc.ibms.sinica.edu.tw, accessed on 10 March 2022) and Liverome (http://liverome.kobic.re.kr/index.php, accessed on 10 March 2022) databases [1,26,27].

### 4.4. Intersecting Target Identification

The VENNY 2.1 online tool (https://bioinfogp.cnb.csic.es/tools/venny/, accessed on 11 March 2022) was used to identify intersected targets between the potential protein targets of active phytochemicals in *Cnidium monnieri* and HCC-related genes [28].

### 4.5. Protein–Protein Interaction (PPI) Analysis

The identified intersected targets (Section 2.4) were then screened for PPI analysis with a high confidence score of 0.700 and species limited to “Homo sapiens” utilizing the STRING database (https://string-db.org/, version 11.5, accessed on 11 March 2022) [29]. The outcomes of the PPI analysis were further envisioned by employing the Cytoscape software (version 3.9.0, Boston, MA, USA, accessed on 11 March 2022) to determine the potential anti-HCC core targets [30].

### 4.6. Network Construction of Active Phytochemicals and Anti-HCC Targets

We further constructed a network of active phytochemicals and anti-HCC targets (in Section 2.5) with Cytoscape software (version 3.9.0, Boston, MA, USA, accessed on 11 March 2022) to analyze their interaction [30].

### 4.7. Enrichment Analysis

Using the database for annotation, visualization, and integrated discovery (DAVID; Version 6.8) (https://david.ncifcrf.gov/, accessed on 12 March 2022), gene ontology (GO) functional enrichment analysis and Kyoto Encyclopedia of Genes and Genomes (KEGG) pathway enrichment analysis were further carried out on 67 intersected targets (determined in Section 2.4) [32]. The GO terms were categorized into three types: cellular component (CC), biological process (BP), and molecular function (MF). By uploading the data to the Bioinformatics platform (http://www.bioinformatics.com.cn/, accessed on 12 March 2022), the top 10 GO analysis data values (BP, CC, and MF) and top 30 KEGG pathways were further shown in the form of an enrichment dot bubble [59]. The classical hypergeometric test was used to determine statistical significance. The adjusted *p* ≤ 0.05 was utilized as the significance threshold in our investigation.

### 4.8. Molecular Docking

Two-dimensional (2D) structures of key *Cnidium monnieri* active phytochemicals were retrieved in Spatial Data File (SDF) format from the NCBI PubChem (https://pubchem.ncbi.nlm.nih.gov/, accessed on 15 March 2022) online database. Their three-dimensional (3D) structures were created and saved in PDB format using BIOVIA Discovery Studio Visualizer 2021. The Protein Data Bank (https://www.rcsb.org/, accessed on 15 March 2022) was used to obtain the crystal structures of the anti-HCC core targets (EGFR, CASP3, ESR1, MAPK3, ERBB2, and CCND1) [60]. The crystal structure complex’s ligands and water molecules were extracted using BIOVIA Discovery Studio Visualizer 2021 software. Furthermore, this was used to construct the grid and prepare proteins [61]. The PDB files were uploaded to AutoDock Vina (version 1.2.0.), and the receptor proteins were charged with Kollman and Gasteiger partial charges. The key active phytochemicals were then uploaded to AutoDock Vina (version 1.2.0.) in PDB format. AutoDock Vina (version 1.2.0.) was used to convert both proteins and key active phytochemicals to pdbqt format. Subsequently, proteins and key active phytochemicals in pdbqt format were employed to write scripts for molecular docking using AutoDock Vina (version 1.2.0.), and docked complex findings were acquired. [62]. The docked complexes were further analyzed to determine the molecules’ and targets’ binding capabilities using BIOVIA Discovery Studio Visualizer 2021 software [61]. A binding energy < 0 implies that a ligand may instinctively bind to the receptor. It is commonly recognized that the lower the energy score of the ligand and receptor binding configuration, the more probable that binding will occur [62].

## 5. Conclusions

We have successfully investigated the key active phytochemicals and molecular mechanisms of *Cnidium monnieri* implicated in the treatment of HCC. This research identified 8 key active phytochemicals of *Cnidium monnieri* and 25 anti-HCC core targets. Our study showed that protein phosphorylation inhibition, positive regulation of protein kinase B signaling, negative regulation of the apoptotic process, the cytokine-mediated signaling pathway, MAPK cascade, proteolysis, and positive regulation of cell migration are all likely underlying mechanisms of *Cnidium monnieri*’s anti-HCC effects. Furthermore, we found that eight key pathways are likely to be involved: pathways in cancer, proteoglycans in cancer, the PI3K–AKT signaling pathway, estrogen signaling pathway, TNF signaling pathway, VEGF signaling pathway, ErbB signaling pathway, and EGFR tyrosine kinase inhibitor resistance, by which *Cnidium monnieri* treats HCC. Our findings justify the conclusion that the anti-HCC effects of *Cnidium monnieri* may be a consequence of the direct or indirect synergistic effects of multitarget and multi-pathway efforts. Molecular docking results demonstrated that key active phytochemicals of *Cnidium monnieri* can potentially bind to HCC-related targets (EGFR, CASP3, ESR1, MAPK3, CCND1, and ERBB2). Although experimental validation is warranted, the results of the present study offer clues for further investigation of the anti-HCC phytochemicals and mechanisms of *Cnidium monnieri* and provide a basis for developing modern anti-HCC drugs based on phytochemicals that occur in *Cnidium monnieri*.

## Figures and Tables

**Table 1:** Active phytochemicals in *Cnidium monnieri*.

ID	Phytochemical Name	Structure	OB	DL
MOL001510	24-epicampesterol	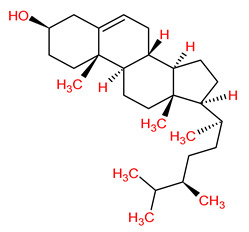	37.58	0.71
MOL001771	Poriferast-5-en-3beta-ol	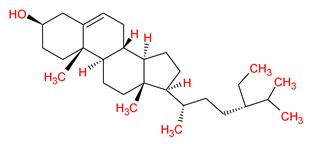	36.91	0.75
MOL001941	Ammidin	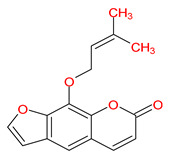	34.55	0.22
MOL002881	Diosmetin	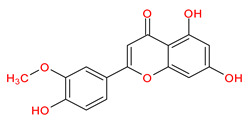	31.14	0.27
MOL002883	Ethyl oleate (NF)	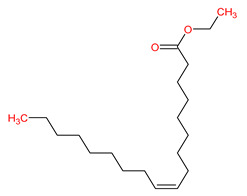	32.40	0.19
MOL000358	Beta-sitosterol	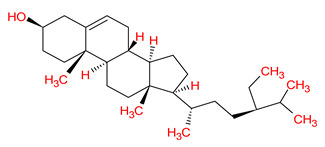	36.91	0.75
MOL003584	Xanthoxylin N	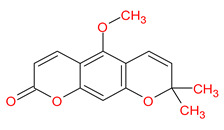	35.51	0.21
MOL003588	Prangenidin	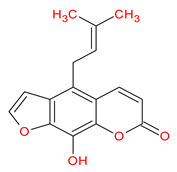	36.31	0.22
MOL003591	Ar-curcumene	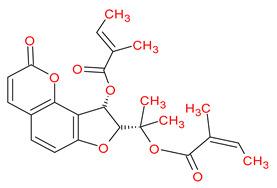	52.34	0.65
MOL003600	Cnidimol B	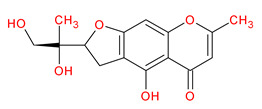	68.66	0.26
MOL003604	Cnidimol F	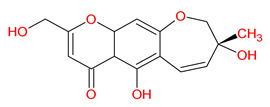	54.43	0.28
MOL003605	(E)-2,3-bis(2-keto-7-methoxy-chromen-8-yl)acrolein	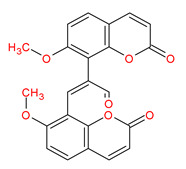	56.38	0.71
MOL003606	Cniforin A	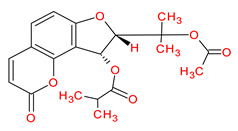	55.89	0.47
MOL003607	Cniforin B	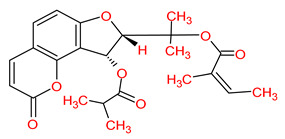	36.70	0.60
MOL003608	*O*-acetylcolumbianetin	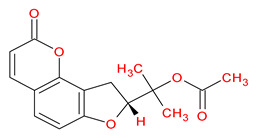	60.04	0.26
MOL003617	Isogosferol	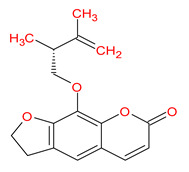	30.07	0.25
MOL003624	*O*-isovalerylcolum bianetin	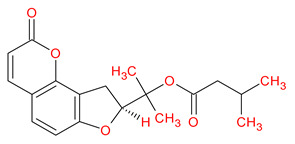	64.03	0.36
MOL003626	Ostruthin	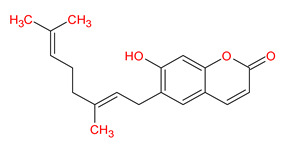	30.65	0.23
MOL000449	Stigmasterol	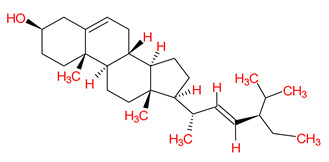	43.83	0.76

**Table 2:** Molecular docking results in terms of the binding energy of eight key active phytochemicals of *Cnidium monnieri* with six anti-HCC core targets.

Molecule ID	Molecule Name	Binding Affinity (kcal/mol)					
		EGFR	CASP3	ESR1	MAPK3	CCND1	ERBB2
MOL003608	*O*-acetylcolumbianetin	−6.9	−5.2	−7.9	−7.3	−7.8	−3.6
MOL002881	Diosmetin	−7.3	−6.2	−8.2	−7.8	−7.3	−3.4
MOL003584	Xanthoxylin N	−6.9	−5.3	−7.7	−7.4	−7.5	−3.4
MOL003591	Ar-curcumene	−8.3	−6.1	−8.4	−7.8	−8.0	−3.5
MOL003606	Cniforin A	−7.2	−5.2	−8.3	−7.6	−7.7	−3.7
MOL003624	*O*-isovalerylcolum Bianetin	−7.5	−5.4	−8.7	−7.2	−7.4	−3.1
MOL003607	Cniforin B	−7.3	−5.7	−9.3	−7.9	−7.9	−3.7
MOL003605	(E)-2,3-bis(2-keto-7-methoxy-chromen-8-yl)acrolein	−8.1	−6.2	−8.0	−8.1	−8.2	−4.3

## Data Availability

Not applicable.
